# A PCR-based method for the diagnosis of *Enterobius vermicularis* in stool samples, specifically designed for clinical application

**DOI:** 10.3389/fmicb.2022.1028988

**Published:** 2022-11-17

**Authors:** Aldo Ummarino, Michele Caputo, Francesco Antonio Tucci, Gaetano Pezzicoli, Ada Piepoli, Annamaria Gentile, Tiziana Latiano, Anna Panza, Nicholas Calà, Antonio Pio Ceglia, Giovanni Pistoio, Vincenzo Troiano, Michela Pucatti, Anna Latiano, Angelo Andriulli, Antonio Tucci, Orazio Palmieri

**Affiliations:** ^1^Agorà Biomedical Sciences, Etromapmacs Pole, Lesina (FG), Italy; ^2^Gastroenterology Unit, Fondazione IRCCS “Casa Sollievo Della Sofferenza” Hospital, Viale Cappuccini, Italy

**Keywords:** *Enterobiasis vermicularis*, PCR, stool (DNA) test, pinworm infection, ribosomal DNA–rDNA

## Abstract

**Background:**

*Enterobius vermicularis* (*E. vermicularis*) is a nematode that infects up to 200 million people worldwide, despite effective medications being available. Conventional diagnostic tests are hindered by low sensitivity and poor patient compliance. Furthermore, no biomolecular techniques are available for clinical application. The aim of this study was to develop a procedure specifically designed for clinical application to detect *E. vermicularis* by means of PCR.

**Materials and methods:**

Two subject groups were taken into account: a group of 27 infected patients and a control group of 27 healthy subjects. A nested-PCR was performed on fecal samples to detect *E. vermicularis*. Due to the intrinsic difficulties of the fecal matrix, several countermeasures were adopted to ensure the efficient performance of the method: (a) a large amount of feces for the extraction process (20 g instead of 200 mg); (b) a combination of chemical and physical treatments to grind the fecal matrix; (c) an additional purification process for the negative samples after the first nested-PCR; and (d) the selection of a very specific target region for the PCR.

**Results:**

Due to the lack of overlap with other organisms, a sequence of the 5S ribosomal DNA (rDNA) spacer region including the tract SL1 was chosen to design appropriate external and internal primers. The first nested-PCR detected *E.vermicularis* in 19/27 samples from infected patients. After further purification, 5/8 of the negative samples resulted positive at the second PCR. Conversely, all the samples from healthy controls resulted negative to both PCRs. Sensitivity and specificity of the method were, respectively, 88.9% and 100%.

**Conclusion:**

The results prove the high diagnostic accuracy of the proposed method, addressing and overcoming the challenges posed by both conventional tests and PCR-based approaches. Therefore, the method can be proposed for clinical application.

## Importance

The authors of this study have designed a method to detect pinworms in human stool samples, specifically for clinical application. The purpose of this method is (a) to help clinicians in the diagnosis and cure of patients with suspected *E. vermicularis* infection, (b) to assess the real epidemiology in the general population and in specific subgroups of subjects, (c) to determine the pathogenetic role of the parasite in some gastrointestinal disorders (opening unexpected clinico-pathological scenarios), and (d) to provide significant support in the extraction and purification of nucleic acids from the feces to other diagnostic methods of parasite detection.

## Introduction

*Enterobius vermicularis* (*E. vermicularis*), commonly referred to as “pinworm,” is a nematode infecting the human intestine. Affecting up to 200 million people worldwide (Panidis et al., 2011), this parasite is widespread all over the world (Gutierrez, 2000), and pinworm infection is the most common infection among helminths in Western Europe and United States (Burkhart and Burkhart, 2005).

Enterobiasis is typically associated with anal itch. However, the infection may also occur in the absence of clinical symptoms and, in a relevant percentage of cases, it can manifest with abdominal pain and/or altered bowel habits, also with no itch (Brewster, 1989; Wu et al., 2000; Brown, 2006; Jardine et al., 2006; Rajamanickam et al., 2009).

The high prevalence of *E. vermicularis* infection suggests its potential involvement in the pathogenesis of different conditions. Indeed, abdominal discomfort or pain and altered bowel habits are highly frequent symptoms in irritable bowel syndrome (IBS). In addition, the eradication of the parasite in patients with both IBS and evidence of *E. vermicularis* infection has cured the symptoms in some cases (Wu et al., 2000; Petro et al., 2005).

Similarly, the pathogenesis of recurrent abdominal pain in children is unclear, and intestinal infection by nematodes has often been considered in this setting (Jardine et al., 2006).

Infection by *E. vermicularis* has finally been held responsible for the peripheral eosinophilia in some patients with no clear underlying causes (Schroeder et al., 2019), as well as for several cases of misdiagnosed acute appendicitis (Panidis et al., 2011; Risio et al., 2016; Dunphy et al., 2017; Sosin et al., 2019).

Unfortunately, the lack of reliable tests for parasite detection does not allow neither for the estimation of the exact role of *E. vermicularis* in all these conditions nor for the actual infection prevalence.

The only two currently available diagnostic tests (adhesive tape test and parasitological test) are, in fact, characterized by low sensitivity, which may drop to 5–15% (Cook, 1994; Jardine et al., 2006). Consequently, infection by *E. vermicularis* remains undetected in a relevant number of cases and enterobiasis control remains difficult, despite the availability of medications for decades (Wendt et al., 2019).

This bottleneck could be eliminated through the use of a high sensitivity diagnostic molecular biology technique. The PCR (Polymerase Chain Reaction) may be taken into consideration, provided that the parasite detection is performed in stool samples, as they represent the main and most practical biological source material available for clinical practice.

However, designing such a test is hindered by the difficult matrix of feces, known for the presence of many PCR inhibitors (Rossen et al., 1992; Tebbe and Vahjen, 1993; Abu Al-Soud and Rådström, 2000; Morin et al., 2001; Mavziutov et al., 2003; Eggert et al., 2005). For this reason, adequate DNA extraction and purification methods are highly necessary.

To date, several reports (Liu et al., 1995; Blouin, 2002; Iñiguez et al., 2002; Floyd et al., 2005; Nakano et al., 2006; Leles et al., 2009; Khouja et al., 2010; Piperaki et al., 2011; Zelck et al., 2011; Ferrero et al., 2013; Ngui et al., 2014; Sow et al., 2017; Tomanakan et al., 2018; Köller et al., 2020; Medkour et al., 2020) have taken into account the use of PCR for the characterization of *E. vermicularis* (Table 1). However, almost none of the studies were specifically designed to develop a diagnostic method for clinical application. As a matter of fact, these studies were carried out with philological, taxonomic, or archeological purposes, with target regions intentionally not highly specific (e.g., mitochondrial DNA). On the contrary, for diagnostic purposes, high conservation in the species is desirable.

**Table 1:** Studies that used the PCR analysis for the characterization of *E. vermicularis*.

Article	Aim of the study	DNA source	Target sequence	Control group
Tomanakan et al. (2018)	To analyzes the genetic diversity of *E. vermicularis*.	Eggs	cox1, ITS2	no
Zelck et al. (2011)	To perform a genetical characterization of pinworms from different regions of Germany.	Worms	ITS1, ITS2, 18S, 5.8S	no
Piperaki et al. (2011)	To investigate the genetic variation within *E. vermicularis* in humans.	Eggs	cox1	no
Medkour et al. (2020)	To perform a survey of parasites using a fast-typing technique by PCR in feces, to assess potential zoonotic transmission.	Feces	5S rDNA region	no
Ferrero et al. (2013)	To perform a genetic study of pinworms in Denmark with DNA extracted from individual eggs.	Eggs	cox1	no
Iñiguez et al. (2002)	To investigate the genetic variation within *E. vermicularis* in humans.	Feces	SL1	no
Liu et al. (1995)	Case report of a hemorrhagic eosinophilic enterocolitis associated with *E. vermicularis*.	Worms	28S rDNA, 5SrDNA spacer region	no
Floyd et al. (2005)	To design PCR primers to amplify a c. 1 kb fragment of the 18S ribosomal DNA gene (specific to the phylum Nematoda).	Worms	18s rRNA gene	no
Leles et al. (2009)	To introduce a method that would allow molecular diagnosis of *Ascaris* sp. from feces as an alternative source of *Ascaris* sp. material.	Feces, eggs	ITS1, mtDNA (citocrome B gene)	no
Khouja et al. (2010)	To determine the presence of Giardia cysts and Cryptosporidium oocysts in raw and treated wastewater, and to sequence-characterize samples.	Wastewater	SSU rDNA	no
Sow et al. (2017)	To compare the performance of real-time PCR assays to microscopic examination for detection of intestinal parasites.	Feces	5S rDNA	yes *
Blouin (2002)	To compare DNA sequence divergence at ITS-1 and ITS-2 with divergence at mitochondrial cox1 or nad4 loci.	Worms	ITS1, ITS2, cox1	no
Nakano et al. (2006)	To analyze sequences of cox1 gene, ITS2 and 5S rDNA of *E. vermicularis* from chimpanzees and to compare them with those of pinworm eggs from humans.	Worms, eggs	cox1	no
Köller et al. (2020)	To assess and compare the performance of different diagnostic qPCR approaches for human parasites and microsporidia in stool samples without a gold standard.	Feces	Cox1, 28S rDNA, 18S rDNA	no
Ngui et al. (2014)	Case report of an invasive *E. vermicularis* infection in a fallopian tube.	Tissue sections	5-subunit rDNA (5S rDNA)	no

*Subjects included in the control group having only a negative parasitological test (which is notorious for having a very low sensitivity).

In addition, the PCR assays of these investigations often rely on worm and egg isolation, which are difficult to obtain from infected patients, thus unlikely to be recommended for clinical practice.

Finally, no validation tests confirming the diagnostic validity of the methods implemented were included in these reports. In particular, the assessment with a negative control group was missing in almost all cases or was not adequate (Sow et al., 2017).

The purpose of the present study was to develop a PCR based method to detect *E. vermicularis* in stool samples for clinical application, overcoming the limitations of the conventional tests (low sensitivity and poor patient compliance) and those of the other molecular approaches.

## Materials and methods

### Study population

Two different groups of subjects were tested: patients with *E. vermicularis* infection and healthy patients.

#### Patients with *Enterobius vermicularis* infection

Twenty-seven patients (13 males, 14 females; 38 ± 19 years, m ± SD, all Caucasians) with proven infection were included. The criteria used to determine the presence of pinworms were: (1) evidence of the parasite in the feces (evaluated by stereomicroscopic identification) and (2) evidence of characteristic pinworm eggs on adhesive tapes (identified by light microscopy). Patients who met one or both criteria were considered positive to *E. vermicularis* infection. All these patients manifested anal itch as the main symptom of the infection.

#### Healthy controls

Twenty-seven age and sex matched subjects (13 males, 14 females; 39 ± 14 years, m ± SD, all Caucasians) were chosen for the control group. The study inclusion criteria were: (1) no evidence of *E. vermicularis* in both adhesive tape test and parasitological analysis; (2) no reported evidence of worms in feces; (3) absence of anal itch; (4) no past history of previous *E. vermicularis* infection; (5) no family history of *E. vermicularis* infection; (6) absence of abdominal discomfort (pain, meteorism, etc.); (7) absence of altered bowel habits; and (8) no promiscuity with children (primary school teachers, pediatricians, baby sitters, nannies, etc. were excluded).

#### Ethical approval

All procedures were carried out in accordance with the ethical standards of the Institutional Committee *Casa Sollievo della Sofferenza Hospital* (132 CE/2015), and with the 1964 Declaration of Helsinki declaration and its subsequent amendments or comparable ethical standards. Informed consent was signed by all participants.

### Stool samples

**Figure 1:**
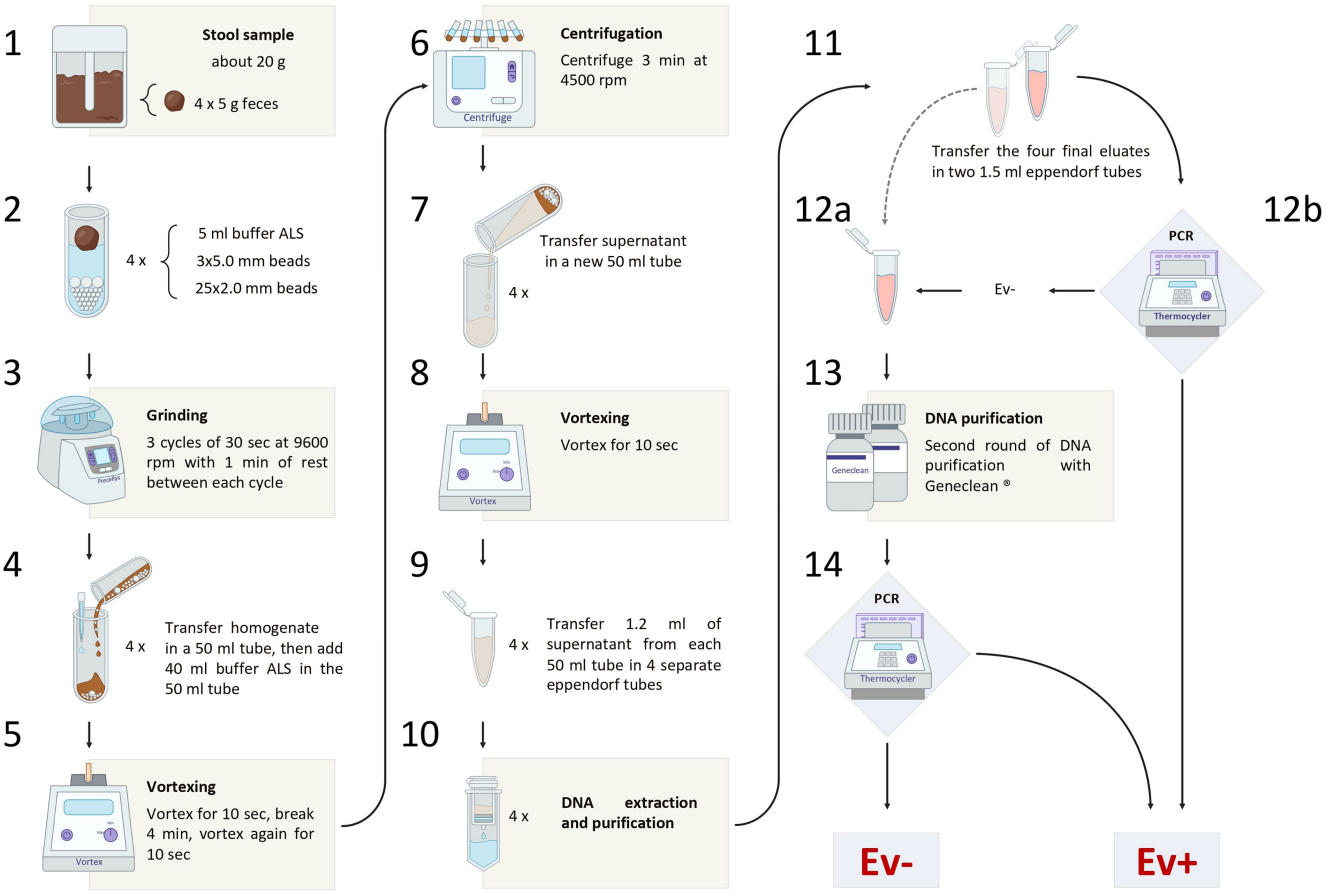
The figure shows the entire sample processing workflow, from the sample collection to the final diagnosis of *Enterobius vermicularis* infection.

The entire sample processing workflow is shown in Figure 1. A stool sample of about 20 g was obtained from each subject of both groups. The samples were collected in sterile containers and stored at −20°C, until processing.

#### Sample preparation

Four aliquots of 5 g were obtained from each defrosted stool sample. Each aliquot was transferred in a 15 ml tube containing a mix of zirconia beads (25 of 2.0 mm diameter and 3 of 5.0 mm diameter).

Five milliliters of buffer ASL (QIAamp DNA Stool Mini Kit, Qiagen, Hilden, Germany) were added to each tube. Then, all tubes were subjected to a grinding process, using Precellys Evolution Homogenizer (Bertin Instruments, Montigny-le-Bretonneux, France).

A set of 3 cycles of 30 s each, at 9,600 rpm, was carried out, with a resting time of 1 min at room temperature between each cycle. The resulting homogenate, together with the beads, was transferred into four 50 ml tubes and a further 40 ml of buffer ASL was added to each of them.

The samples were then vortexed for 10 s, incubated at room temperature for 4 min, vortexed for 10 s, and finally centrifuged for 3 min at 4,500 rpm.

Subsequently, the supernatant was transferred into a new 50 ml tube and vortexed for 10 s. Then, 1.2 ml of the supernatant were taken from each of the four 50 ml tubes and transferred into four 1.5 ml eppendorf tubes (for DNA extraction and purification).

#### DNA extraction and purification

Isolation of DNA was performed using QIAamp Stool Mini Kit (Qiagen, Hilden, Germany), according to the “Stool Pathogen Detection” manufacturer’s protocol with the following modifications: (1) the first five steps of the procedure were replaced by the chemo-physical grinding process as described in paragraph “Sample preparation” of the manufacturer’s protocol; (2) the final elution step was performed using half of the suggested buffer volume to increase the DNA final concentration.

The four final eluates obtained from each patient/control were mixed in a single tube, resulting in a cumulative volume of 400 μl.

This pooled eluate was, then, equally divided into two separate 1.5 ml eppendorf tubes. The first tube was used for PCR assay. The second tube, instead, was stored at −20°C, and reserved for further DNA purification to be applied to the samples negative to the PCR assay. Geneclean II Kit (MP Biomedicals, Qbiogene, Inc., Carlsbad, California, United States) was used in this process, according to the manufacturer’s protocol. Thereafter, these samples were retested by PCR assay.

### Target sequence

The 5S ribosomal DNA (rDNA) spacer region has been chosen as target DNA for the identification of *E. vermicularis*.

**Figure 2:**
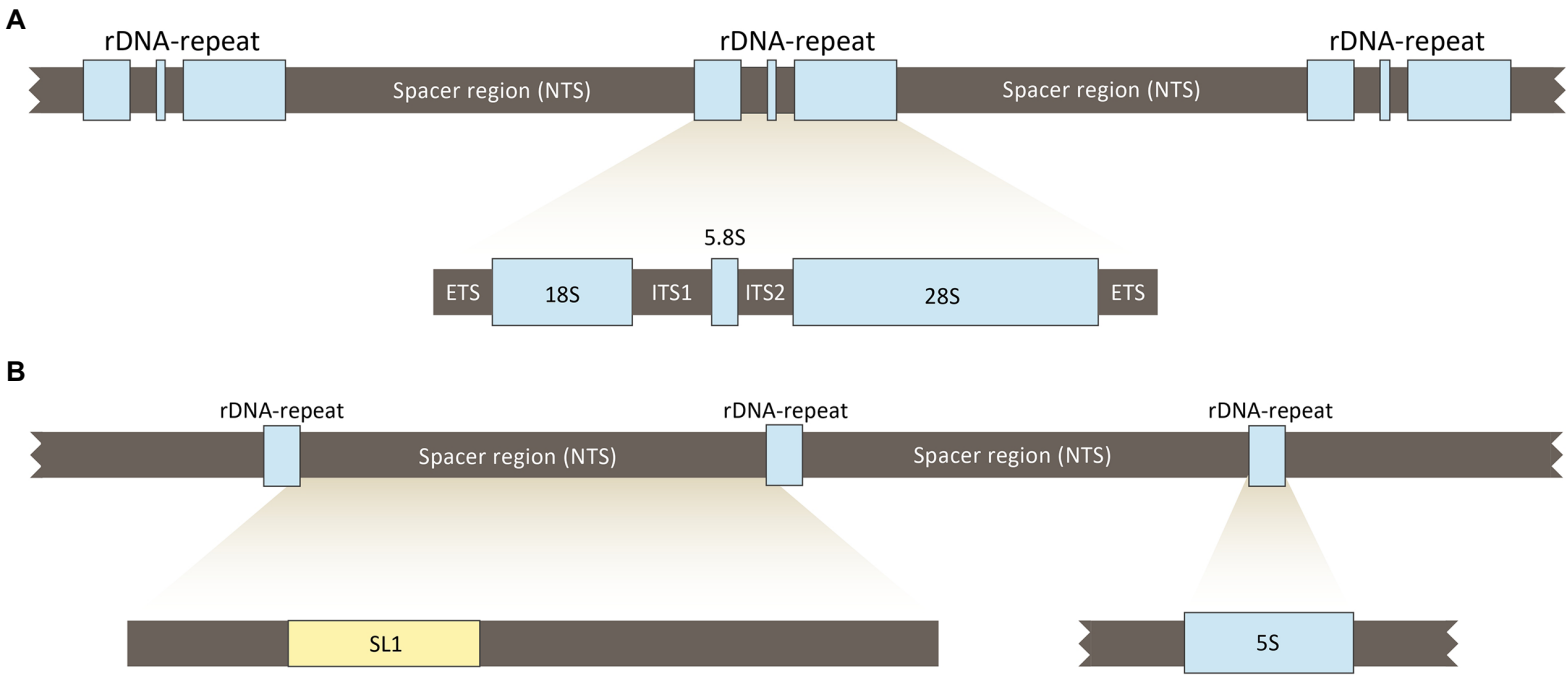
The major and minor multigene families of the ribosomal DNA (rDNA) of *Enterobius vermicularis*. In **(A)** is reported the major family; it is arranged in tandemly repeated clusters (r-DNA-repeat), separated by non-transcribed spacer regions (NTS). In **(B)** is reported the minor gene family; it is also organized in tandem repeated clusters (r-DNA-repeat), separated by non-transcribed spacer regions (NTS). ETS, external transcribed spacers region; ITS, internal transcribed spacers region.

The rDNA is organized into two distinct multigene families, a major and a minor one. The genes of both families are arranged in tandemly repeated clusters containing coding regions, non-transcribed spacer regions (NTS), external, as well as internal spacer regions (ETS and ITS; Figure 2; Richard et al., 2008; Madani et al., 2019; Potapova and Gerton, 2019).

In contrast with the great variability of the spacer regions in other species, *E. vermicularis* shows a highly conserved 5S spacer region (Liu et al., 1996; Iñiguez et al., 2002). This uncommon behavior is due to the selective evolutionary pressure exerted by the spliced leader 1 (SL1) sequence (accession number: AY234778.1),^1^ present in this region, which plays a functional role in the trans-splicing process (Sturm et al., 1999; Xu et al., 2000; Nilsen, 2001; Iñiguez et al., 2006). For this reason, the 5S spacer region spanning over the SL1 subregion was considered as the best target sequence for the purposes of this research study. An 839 bp segment of the 5S spacer region encompassing the entire SL1 was retrieved from the *National Center for Biotechnology Information* (NCBI) database (accession number: AY682469.2)^2^ and analyzed using *Standard Nucleotide BLAST*^3^ to minimize overlaps with other organisms.

### Primer design

*Primer3plus* software (version: Primer3-web 4.0.0)^4^ was employed to build the first set of primer (external). Primers were chosen based on the best settings for a low probability of primer-dimer/hairpin formation, the proper GC content, and the adequate annealing temperature.

The second set of primers (internal) was chosen by considering different options within the product sequence of the 1° round of amplification.

In both cases, the sequences identified by the primers were subjected to further analysis with *Align sequences nucleotide BLAST* (see footnote 3), to verify the specificity of the sequence for the DNA of *E. vermicularis* and the possible overlaps with other organisms.

### Nested PCR

PCR reactions were carried out in a final volume of 50 μl containing GeneAmp 1× Buffer II (10 mM Tris–HCl, pH 8.3; 50 mM KCl), 1.5 mM MgCl_2_, 1.5 U of AmpliTaq Gold DNA polymerase (Applied Biosystems), 22.5 pM of each primer, 20 mM of dNTPs (Promega, Madison, WI, United States), and 5 μl of extracted DNA.

Reactions were performed with initial denaturation at 94°C for 5 min, followed by 40 cycles at 94°C for 1 min, annealing at 56°C for 1 min, and extension at 72°C for 1 min. Final extension was 72°C for 10 min. External primers were used for the first round of amplification, while internal primers for nested PCR starting from 5 μl of the product of the first round and in the same thermocycling conditions.

Five microliters of the product of both the first and the second rounds of PCR were run on a 2% agarose gel containing ethidium bromide and exposed to UV light to visualize DNA bands.

Two electrophoresis markers were used: pGem DNA Marker (Promega, Madison, WI, United States) and 50 bp DNA ladder (Invitrogen, Carlsbad, CA, United States), with two different size scales (36–2,645 bp for pGem and 50–800 bp for 50 bp DNA ladder), to better assess the molecular weight of the amplified sequence.

A positive and negative control was included in each round of the nested PCR experiment.

### Positive and negative control

Two positive controls were obtained by isolating eggs from adhesive tapes of infected patients. Specifically, a small portion (3 mm × 3 mm) of the tape containing eggs, confirmed under microscopic observation, was directly dipped into 100 μl of 0.02 N NaOH solution. As a matter of fact, through this procedure eggs are removed from the adhesive surface of the tape (H. Hasegawa, personal communication, April 20, 2010). Finally, the eggs were subjected to heat shock, consisting of 10 min immersion in 95°C water, followed by 3 min at-80°C. The obtained DNA was stored at −20°C until PCR procedure.

The negative control was obtained by adding 5 μl of ultra-sterile water to the complete PCR mixture.

### Sequencing of PCR products

Amplicons from positive controls were Sanger-sequenced to verify the correspondence with the sequences selected *in silico*. Briefly, PCR products were sequentially purified by GeneClean kit and a vacuum system (Millipore, Bedford, MA, United States), according to the manufacturer’s recommendation.

Purified DNA was then resuspended in 25 μl of dH_2_O and run on 2% agarose gel. Amplicons were sequenced from both ends using an aliquot (3.2 pM) of the PCR reaction primers relying on the BigDye Terminator Cycle Sequencing Kit v. 1.1 (Thermo Fisher Scientific, Somerset, NJ, United States). After purification by using Centri-Sep columns (Princeton separations, Adelphia, NJ, United States), sequencing reactions were loaded on 3500 DX Genetic Analyzer capillaries (Applied Biosystems, Foster City, CA, United States) and analyzed using the Sequencing Analysis software v5.4 (Applied Biosystems, Foster City, CA, United States). The electropherogram results were expressed as the percentage of homology (%). The forward and reverse sequences obtained from each sample were assembled in one sequence using the CAP3 algorithm as implemented in SnapGene software v6.1 (Dotmatics, Boston, MA, United States). Discrepancies between forward and reverse sequences due to poor-quality base calls were manually curated and resolved. The sequences from both the controls were submitted to *NCBI GenBank* (accession numbers: OP650208, OP650209) through the BankIt submission tool and are publicly available.

## Results

### Target sequence

**Figure 3:**
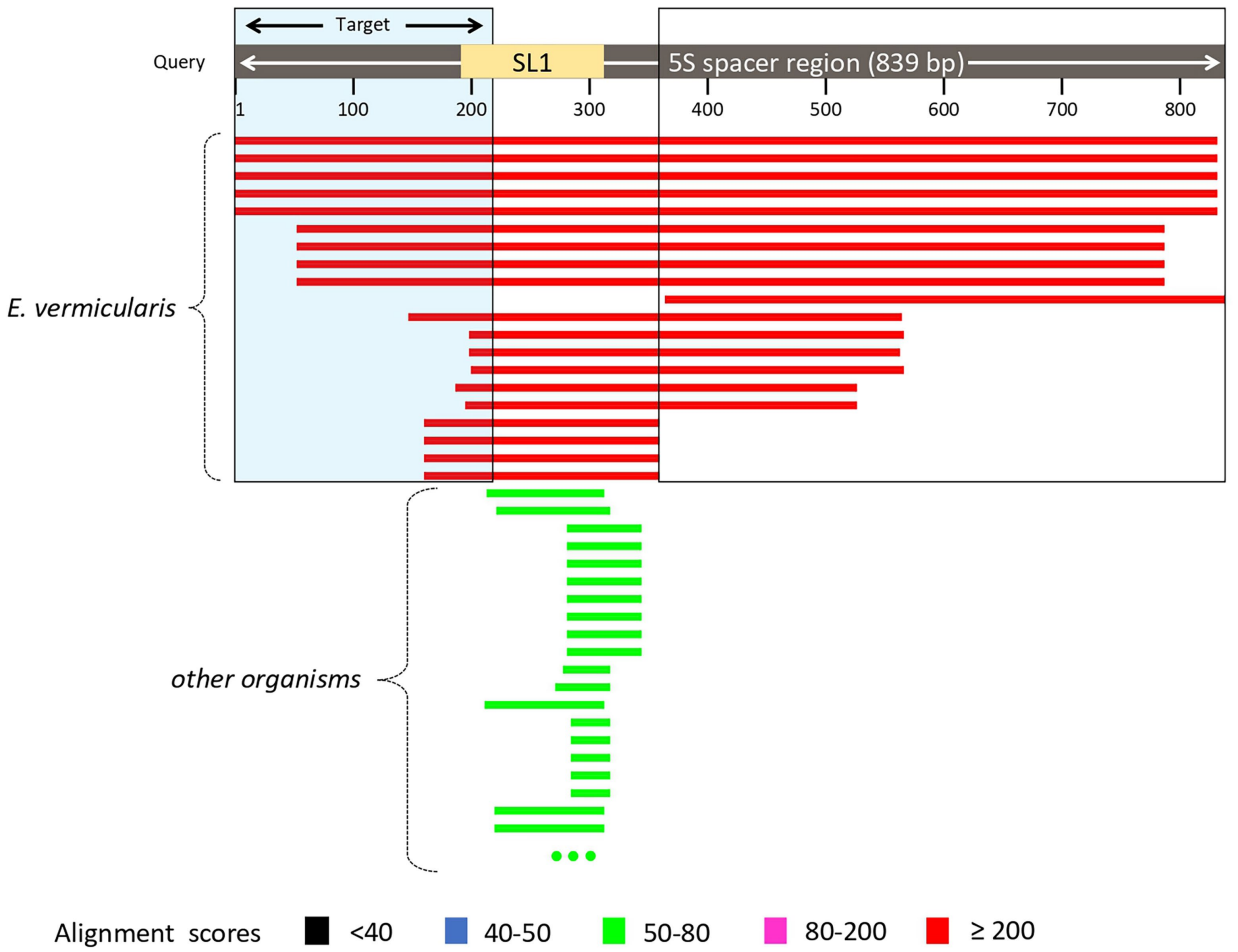
Analysis of *Enterobius vermicularis* SL1 of the 5S spacer region using the Nucleotide Blast tool. The records with the highest alignment score with the query sequence (bars in red) corresponded to different *E. vermicularis* isolates, while those with the lower alignment score (in green), correspond to other organisms. The two segments at both the ends are highly specific for *E. vermicularis*. The first tract corresponds to nucleotides 1–223 (223 bp) and the second one to nucleotides 341–839 (499 bp) of the 5S spacer region.

The analysis of *E. vermicularis* SL1 of the 5S spacer region performed by using the Nucleotide Blast tool in the NCBI database showed that all the records with the highest alignment score with the query sequence (bars in red) corresponded to different *E. vermicularis* sequencings, as expected (Figure 3). However, there were other bars with a lower alignment score (in green) that did not account for *E. vermicularis* and aligned with the middle part of the query sequence in the middle tract, leaving two segments, at both the ends, still highly specific for *E. vermicularis*. The first tract corresponds to nucleotides 1–223 (223 bp) and the second one to nucleotides 341–839 (499 bp) of the 5S spacer region.

This analysis highlighted two candidate regions highly conserved and specific. The first sequence was chosen as target sequence since it partially spanned over the SL1 region and thus is more likely to be conserved within the species.

### Primer design

**Figure 4:**
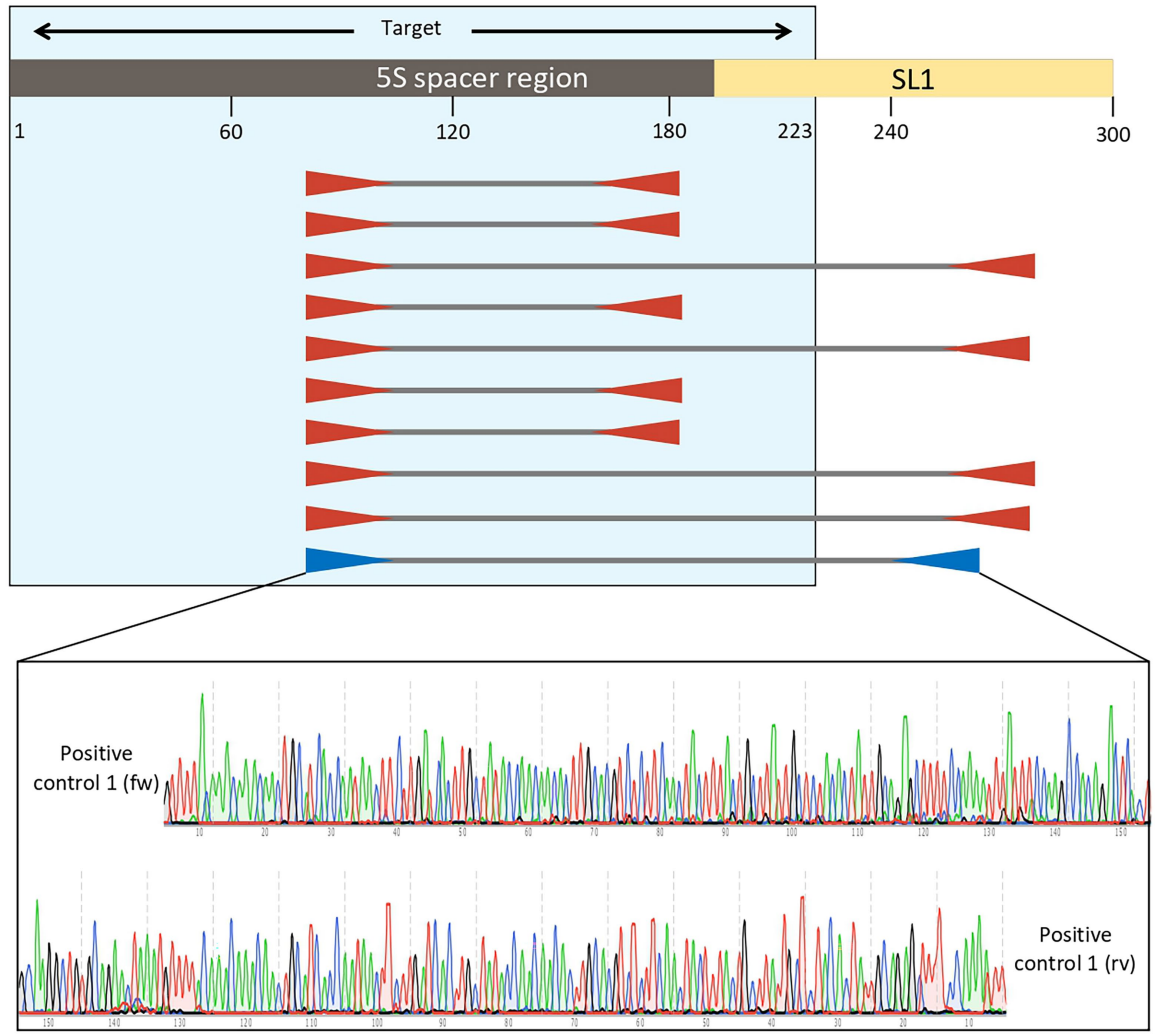
Analysis with *Primer3plus* software using the nucleotides 1–310 of *Enterobius vermicularis* spacer region. The software generates 10 different options of external primers. The couples first, second, fourth, sixth, and seventh do not include the SL1 sequence, while the others do. Among the latter, the tenth couple was chosen for the external primers because it picked the shorter tract (181 bp, nucleotides 82–263) including the target sequence. Assembled reads from Sanger sequencing of the positive control 1 showing perfect homology with the expected *E. vermicularis* amplicon with the selected pairs of primers.

The analysis with *Primer3plus* software was performed using the nucleotides 1–310 of *E. vermicularis* spacer region to provide some spanning around the target sequence and to allow for the design of two couples of nested primers. The software generated 10 different options of external primers (Figure 4). Five couples (first, second, fourth, sixth, and seventh) were discarded as they did not include the SL1 sequence. Among the remaining ones, the tenth couple were chosen because it picked the shorter segment (182 bp, nucleotides 82–263) including the SL1 sequence (Figure 4).

**Figure 5:**
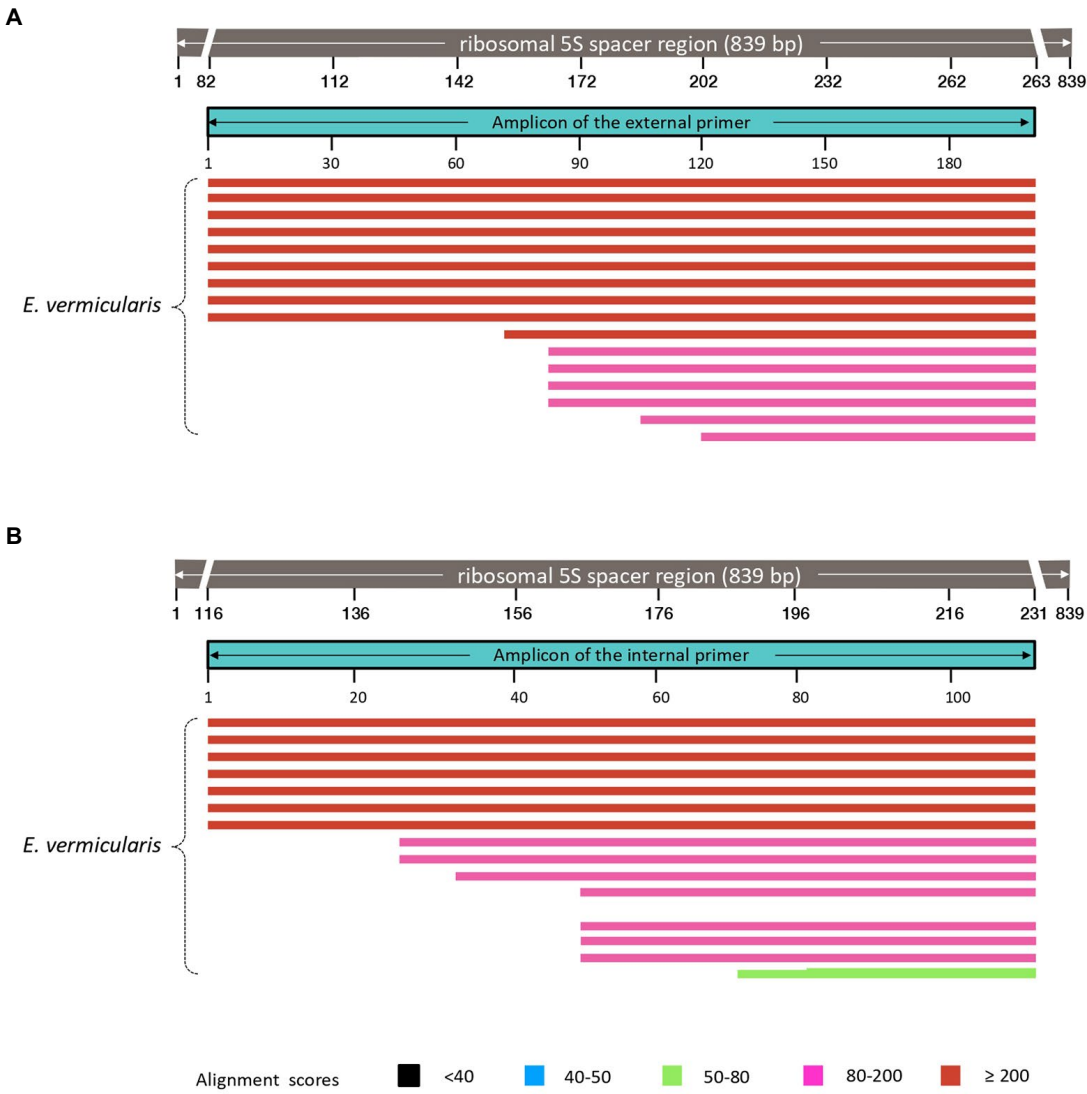
Analysis of the sequences obtained using our primers in the NCBI database. Both external **(A)**, and internal **(B)**, primers select DNA sequences highly specific for *Enterobius vermicularis*, with no cross-reaction with other organisms.

These primers were named “Ev_ext_fw” and “Ev_ext_rv” and their target sequence was further analyzed in the NCBI database, confirming their specificity exclusively for *E. vermicularis* with high alignment scores (Figure 5A).

**Figure 6:**
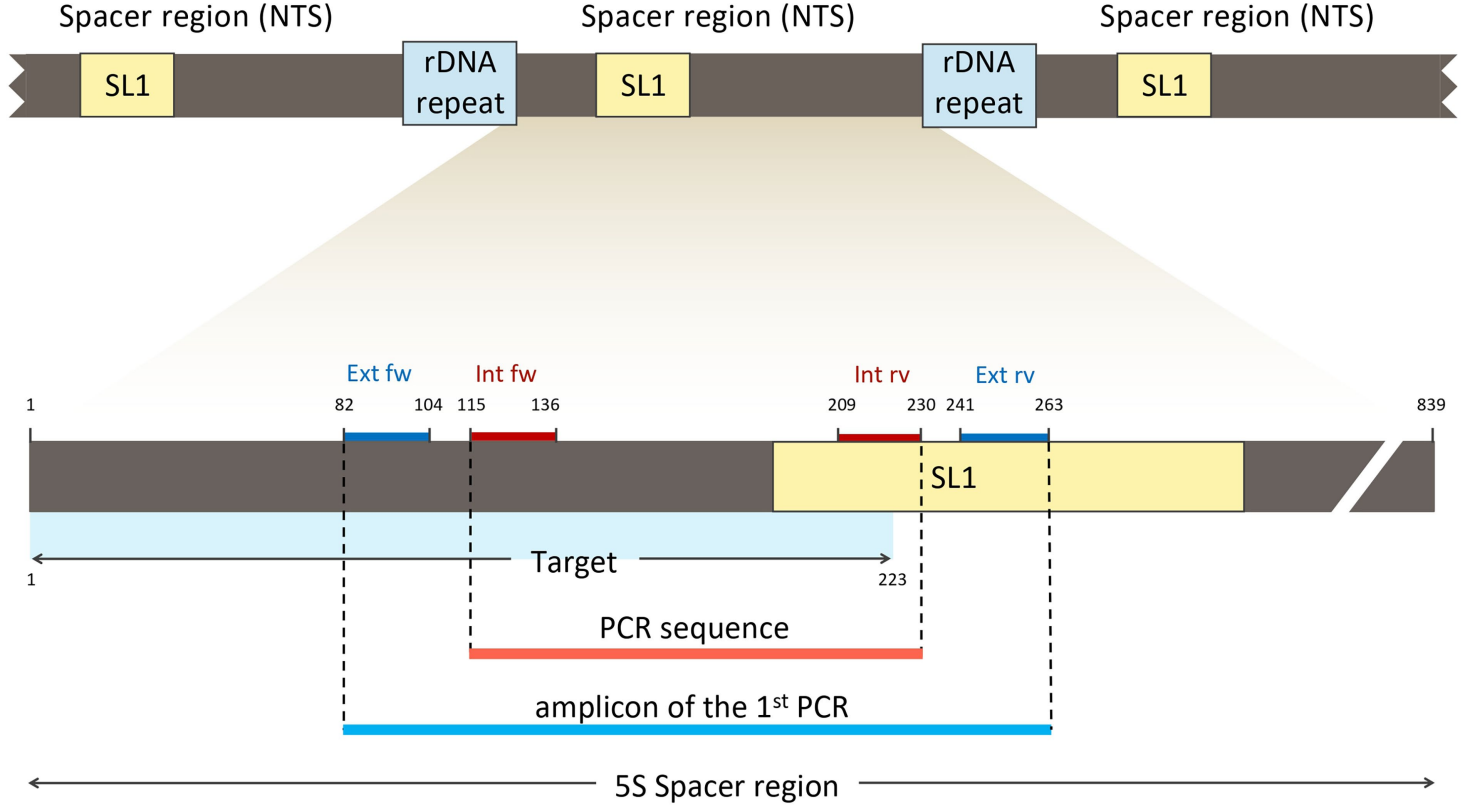
Location of the external (Ext fw and Ext rv) and internal primers (Int fw and Int rv) in relation to the SL1 region and the target sequence.

Next, an inner tract of 116 bp (nucleotides 116–231) was chosen within the amplicon of the first round of amplification to design the second internal set of primers and named them “Ev_int_fw” and “Ev_int_rv.” Also in this case, the analysis in the NCBI database confirmed the specificity of the sequence obtained only for *E. vermicularis* (Figure 5B). The sequences and the characteristics of the primers are summarized in Figure 6 and Table 2.

**Table 2:** External (ext) and internal (int) primers used for the nested PCR.

**Primer**	**Sequence** (5’ ➔ 3’)	**Length** (bp)	**GC content** (%)	**Melting temperature** (°C)
*Ev_ext_fw*	ACAGTGCAAGGCTGTGCAGAACT	23	52.1	69.4
*Ev_ext_rv*	ACATCAGTGAGTCTGTGGCTTGGA	24	50	69.2
*Ev_int_fw*	CAAACAAACAACTGCATCACCA	22	40.9	65.7
*Ev_int_rv*	TGGAAAAGCTCTGCAATAGTGT	22	40.9	62.6

### Positive controls and sequencing of the PCR products

**Figure 7:**
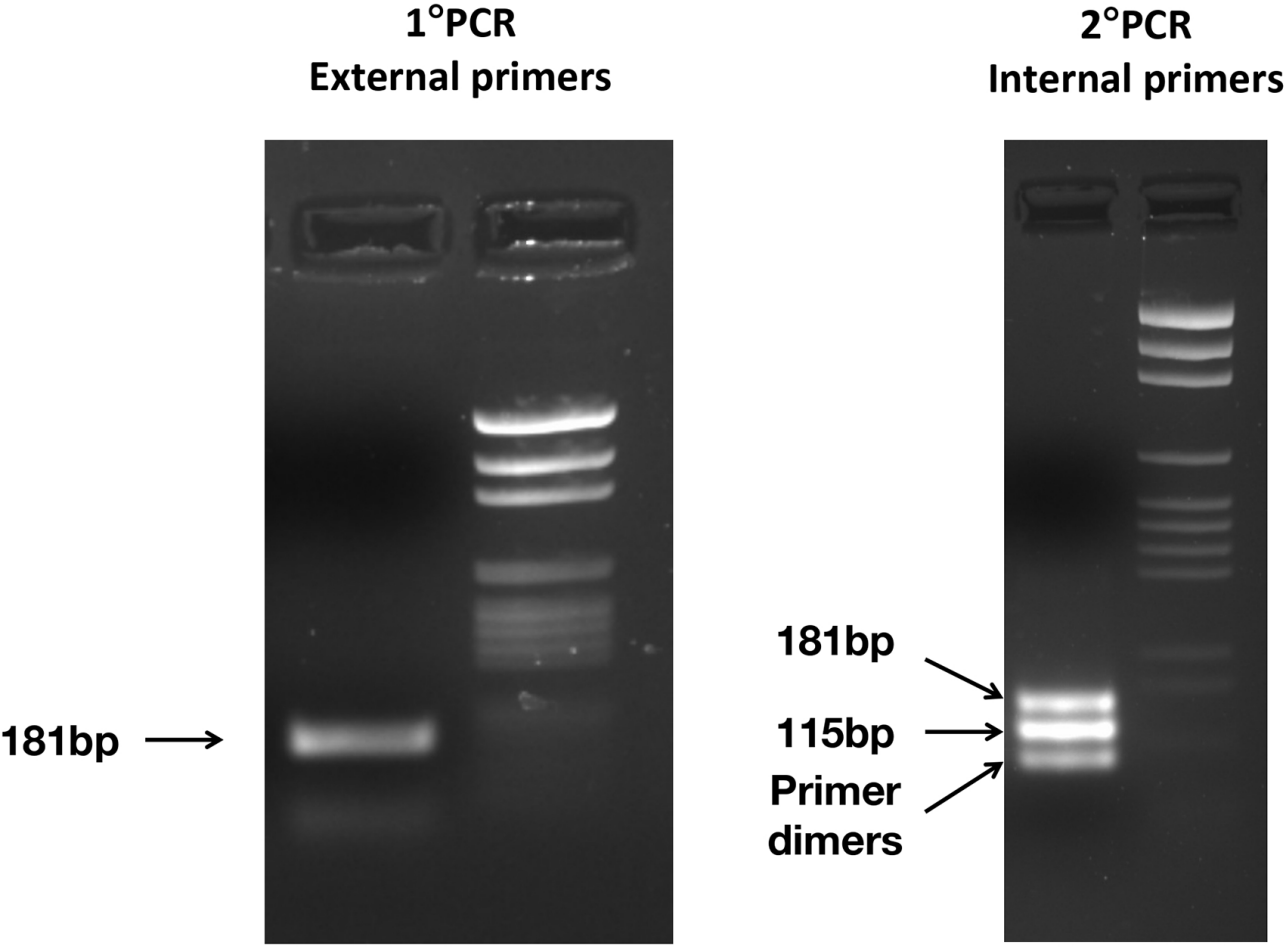
Electrophoresis of the PCR products was obtained after the first PCR (left panel) and the nested PCR (right panel) carried out on one positive control. In the left panel, one band of 181 bp is observed, on the right panel three bands: 182 and 116 bp from the nested PCR, while the lower band from primer dimers.

The nested PCR on the two positive controls obtained from *E. vermicularis* eggs and subsequent agarose gel electrophoresis led to the display of three different bands. The 182 bp upper band is to be traced to the primer’s excess of the first round of amplification; the 116 bp middle band is consequent to the nested PCR; and the lower band is due to dimer primers (Figure 7).

The amplicons obtained after the first round of amplification were Sanger-sequenced with both the forward and reverse primers. We compared the assembled sequences obtained demonstrating a 100% homology with those predicted *in silico* for both samples (Table 3).

**Table 3:** Assembled sequences obtained from DNA sequencing of the amplicons of the first PCR of two different positive controls (CTR1 and CTR2) and comparison with the expected sequence.

*Sample*	Reference sequence (182 bp)	Sequence obtained after sequencing	% query cover (% homology)
*CTR1* (*172 bp*)	ACAGTGCAAGGCTGTGCAGAACTAAATGTTTTACAAACAAACAACTGCATCACCAATAACTTCTTGATCACTTGCTATACCAACAACACTTGCACGTCTCTTCAACTACTTTACTGCTTATTGCTCTACACTATTGCAGAGCTTTTCCAAAATTTATTTCCAAGCCACAGACTCACTGATGT	GCAAGGCTGTGCAGAACTAAATGTTTTACAAACAAACAACTGCATCACCAATAACTTCTTGATCACTTGCTATACCAACAACACTTGCACGTCTCTTCAACTACTTTACTGCTTATTGCTCTACACTATTGCAGAGCTTTTCCAAAATTTATTTCCAAGCCACAGACTCACT	95% (100%)
*CTR2* (*173 bp*)		GGCTGTGCAGAACTAAATGTTTTACAAACAAACAACTGCATCACCAATAACTTCTTGATCACTTGCTATACCAACAACACTTGCACGTCTCTTCAACTACTTTACTGCTTATTGCTCTACACTATTGCAGAGCTTTTCCAAAATTTATTTCCAAGCCACAGACTCACTGATGT	95% (100%)

### Nested PCR in the study population

**Figure 8:**
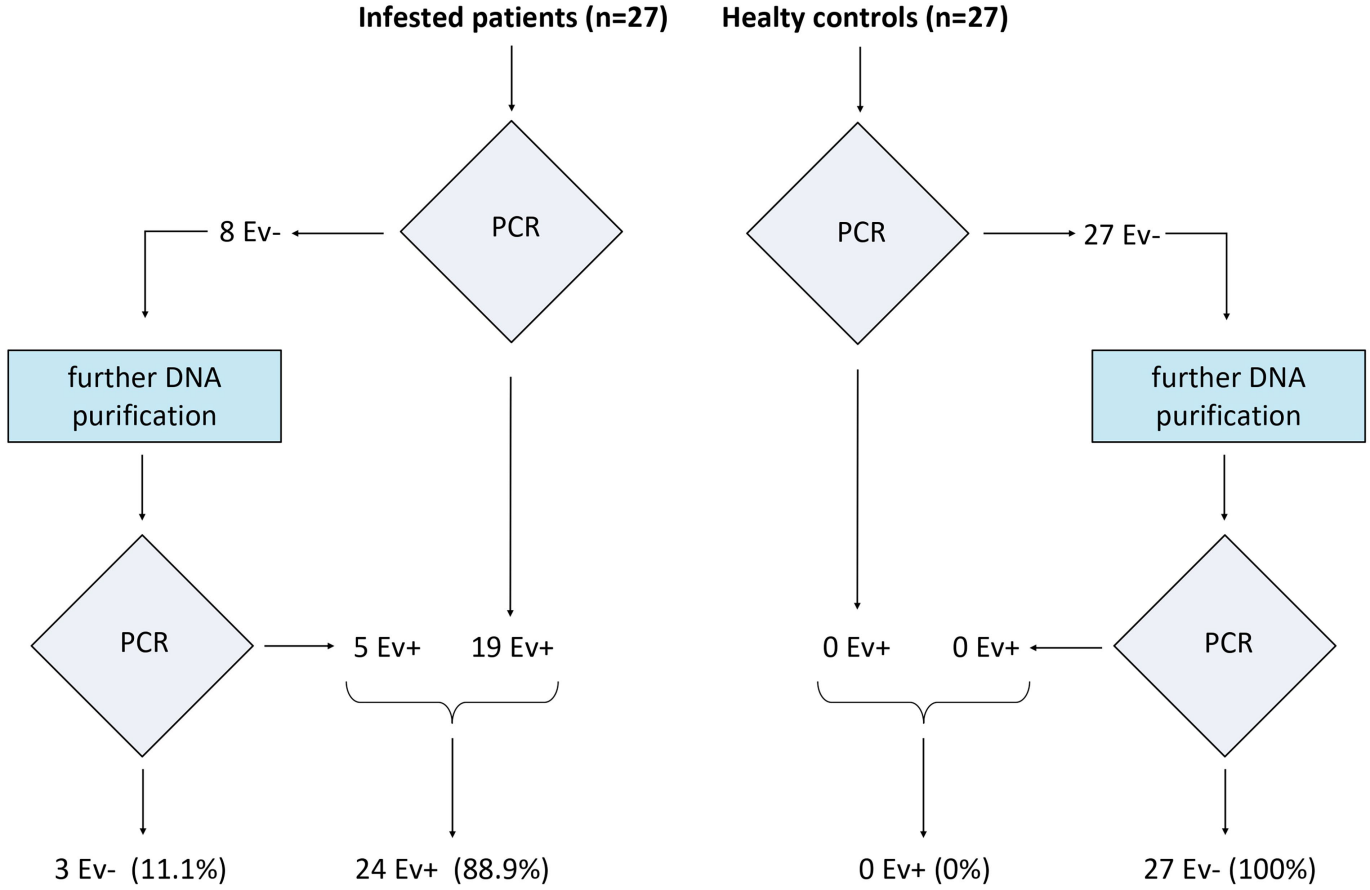
Nested PCR in the study population. On the left are reported the results in the infested patients, while on the right there are those in healthy controls.

*Enterobius vermicularis* was detected by nested PCR in 19 of the 27 extracted DNA samples from infected patients. Among the eight negative patients, the further purification process with GeneClean yielded five additional positive results. Overall, 24 of the 27 infected patients (88.9%) resulted positive with the applied procedures. In healthy controls, nested PCR did not show any evidence of the *E. vermicularis* genome, both in extracted and further purified DNA samples (Figure 8).

Hence, the sensitivity and specificity of the proposed method were 88.9% and 100%, respectively.

## Discussion

Intestinal infection by *E. vermicularis* is one of the most common helminth infections in the world, with up to 200 million infections estimated worldwide (Panidis et al., 2011). Enterobiasis may occur silently, but may often manifest with anal itch and, in some cases, with abdominal pain/discomfort, or altered bowel habits (Brewster, 1989; Wu et al., 2000; Arca et al., 2004; Brown, 2006; Jardine et al., 2006; Rajamanickam et al., 2009). This raises the question on the role of the parasite in recurrent abdominal pain in children and in irritable bowel syndrome.

However, due to the low sensitivity of the tests currently available in clinical laboratories (adhesive tape test and parasitological test; Cook, 1994; Jardine et al., 2006; Remm and Remm, 2009), the actual role of the pinworms in the pathogenesis of these conditions cannot be assessed. For this reason, patients with symptoms caused by the parasite and negative to the relative clinical tests have no access to the medications to treat their condition.

In this study, a PCR-based assay for the diagnosis of enterobiasis, specifically designed for clinical application, has been introduced. One of the major difficulties encountered in the development of such an approach was the DNA isolation and purification from fecal samples. Due to the high amount of PCR inhibitors in the fecal matrix and to the very low amount of the parasite DNA in feces (Monteiro et al., 1997; Abu Al-Soud and Rådström, 2000), the development of a reliable DNA isolation procedure has been quite challenging. Appropriate countermeasures have been adopted to overcome the above limitations and the approach here developed represents one of the most remarkable findings of the authors’ work. Three strategies have been adopted: (a) the use of a large amount of feces for the extraction process (20 g instead of the 200 mg commonly processed in stool PCR testing), (b) the use of a combination of chemical and physical treatments to thoroughly grind the complex fecal matrix; and (c) the adjunct of an additional purification process for the samples resulted negative after the first nested PCR. In our experience, the integration of the three procedures above reported is mandatory for a proper diagnostic tool for the detection of parasites in stool samples.

The choice of using stools as a DNA source for the PCR analysis is the distinctive feature of this study compared to most of the previous studies. Indeed, almost all the PCR reports on *E. vermicularis* employed pinworms or their eggs as source material (Table 1), as these studies were essentially driven by philological, taxonomic, or archeological purposes. However, by doing so, the issue of DNA extraction from feces was simply disregarded resulting in a serious methodological problem. On the other hand, a diagnostic test intended for clinical use based on worm and egg isolation would entail the same as the adhesive tape test. Therefore, the choice of using fecal samples, in our study, is to be considered the most appropriate and in line with the purposes of clinical application, since it requires only a simple stool sample, which is easily obtainable for patients.

In addition to that, further clarification is needed with reference to the choice of the target region. The DNA regions commonly targeted for PCR investigations on parasites are essentially two: the mitochondrial DNA (mtDNA) and the ribosomal DNA (rDNA). Since the former is characterized by a high variability, the focus of this study was on DNA coding for ribosomal subunits, largely conserved and remarkably similar in organisms of the same species. In addition, most of the previous reports, designed for phylogenetic, taxonomic, or archeological purposes, did not prevent cross-reactions with other nematodes (which were even sought in some cases). The primers adopted in the aforementioned studies are, often, general nematode primers, since the aim of these investigations was to evaluate the parasite genetic variability of the parasite and its evolutionary significance within the nematode phylogenetic framework. In contrast, since the test outcome affects both clinical interpretation and therapeutic approach, for clinical purposes a high degree of specificity is required for clinical purposes. For this reason, in the present study, careful attention was paid to the selection of the target sequence. After several investigations, a region of the 5S rDNA spanning over the SL1 subregion was chosen, which proved to be an appropriate solution. As a matter of fact, the simulation analysis performed on Nucleotide BLAST software demonstrated that the high specificity of the target sequence chosen to detect *E. vermicularis*, showing no overlap with sequences of other organisms. Furthermore, the designed primers proved to be reliable and effective in amplifying the target sequence. The performance tests carried out on the software platform and corroborated by the *in vitro* molecular analysis confirm that the sequences of the PCR products (both after the first and the nested PCR) are highly specific and exclusive for *E. vermicularis*. These outcomes are in line with the design philosophy and further support the results obtained in the subject groups investigated. The high positivity rate in the positive control group (88.9%) and the complete absence of *E. vermicularis* positivity among the negative controls (100%), strongly support high specificity, and the overall high diagnostic accuracy of the method. The implementation of a rigorously selected and sex-age matched negative control group in the study significantly contributed to the accuracy and robustness of the results. In all the previous studies reported in literature (Table 1), the assessment in a negative control group was missing in nearly all cases, or, when present, the same was not adequate (i.e., the report of Sow D. et al. that included a negative control group of unselected subjects with only a negative parasitological test, which is notorious characterized by low sensitivity; Sow et al., 2017). However, although rigorous, the authors are aware that the number of subjects enrolled in this study is limited and they think that further investigations in bigger cohorts are needed to corroborate their data. To the authors’ best knowledge, there is only one kit available to diagnose *E. vermicularis* infestation through real-time PCR (RT-PCR) on feces (AmpliTest *Enterobius vermicularis*, Amplicon, Wroclaw, Poland). However, up to date, no specific data have been reported, no validation tests have been presented, and no publications have been published in the literature regarding this kit. Another kit (Allplex™ GI-Helminth assay) is also available for helminths detection through PCR, but a recent publication demonstrated low specificity (36% of false negative results in the cohort investigated) in the diagnosis of *E. vermicularis* in stool samples (Autier et al., 2021).

Currently, the scotch tape technique is considered the gold standard for *E. vermicularis* detection. However, it is widely known that, when it comes to very high diagnostic specificity, its sensitivity is very low (Cook, 1994; Jardine et al., 2006; Remm and Remm, 2009) and up to three tests in three different consecutive days are required to increase the chances of confirming the diagnosis (Wendt et al., 2019). This lack of sensitivity is due to the parasite vital cycle, which inhabits the terminal ileum-cecum-appendix region and only occasionally migrates into the rectum, laying eggs in the perianal region.

A similar scenario occurred in the recent past with *Helicobacter pylori* (*H. pylori*). In the last decade of 1900 and in the first decade thereafter, the gold standard for the diagnosis of the infection was the microbial culture (Mégraud, 1995). As with the scotch test, microbial culture reported high specificity but a low sensitivity. For years, the same was considered the reference (gold standard) for the diagnosis. However, it has been later replaced by the urea breath test, which is more sensitive and only slightly less specific (Pohl et al., 2019).

With regard to *E. vermicularis* there is the need for a specific and sensitive method, and, showing 88.9% sensitivity and 100% specificity, the method implemented in the present study seems to meet this condition. Certainly, it has two undeniable intrinsic limits: complexity and costs. However, if on the one hand, these limits cannot be underestimated, on the other hand, they are justified by the constraints of the research implementing a method with not only a high sensitivity and diagnostic specificity, but also non-invasive and easy to perform on patients. The collection of 20 g of feces (less than a plum) for a patient does not represent a difficulty, in light of the small amount considered, and it can easily fit in a normal container for feces or urine.

As for its complexity, as in the case of *H. pylori*, new methods are usually initially complicated, and then progressively simplified and economized (i.e., the first devices for the urea breath test were cumbersome, complex, very expensive, and used radioactive reagents; the current ones are small, simple, cheap, and do not use radioactive components). Therefore, the authors believe that further investigations will lead to a simplified, more affordable, and improved method compared to the procedure described in the present study.

However, regardless of possible future developments, this research is undoubtedly to be credited for: (1) the design of a method to detect *E. vermicularis*, easy to perform on patients; (2) the identification of a highly specific target region for *E. vermicularis* preventing cross-reactions with other nematodes; (3) the development of a strategy for the isolation and purification of DNA parasite from difficult fecal matrix; (4) the implementation of a diagnostic method characterized not only by a good specificity, but also by a high sensitivity; and (5) the validation of the proposed method with a carefully selected sample of positive and (especially) negative controls.

In light of these considerations, a relevant impact of the findings reported on future research is to be hypothesized. Firstly, the proposed method, if validated in larger cohorts of patients and simplified in the procedure, could access clinical practice as the main tool for the diagnosis of *E. vermicularis,* thus becoming the gold standard. This would help the clinicians to correctly interpret, and consequently treat, symptom profiles in patients with suspected enterobiasis.

Secondly, such a test could be used to assess the actual prevalence of *E. vermicularis* in the general population and in specific subgroups of subjects.

Thirdly, the new diagnostic tool could be used to determine the pathogenetic role of the parasite in some gastrointestinal disorders (such as irritable bowel syndrome and recurrent abdominal pain in children) and may even open unexpected clinico-pathological scenarios.

Finally, the nucleic acid isolation and purification processes developed for the detection of *E. vermicularis* can be taken from other diagnostic methods for the detection of other parasites in feces.

## Data availability statement

The original contributions presented in the study are included in the article/Supplementary material, further inquiries can be directed to the corresponding author.

## Ethics statement

The studies involving human participants were reviewed and approved by Casa Sollievo della Sofferenza Hospital (132 CE/2015). The patients/participants provided their written informed consent to participate in this study.

## Author contributions

AU performed most of the experiments, wrote part of the manuscript, and contributed to the overall organization of the paper. MC, GiP, TL, APa, NC, AC, GaP, VT, MP, and AG contributed to the experiments, especially in the extraction of the DNA from the human specimens, in the PCR assays, and in the sequencing reactions. FAT contributed to the graphics and to the manuscript. APi, AA, AL, OP, and AT supervised the writing of the manuscript and edited the materials overall. All authors contributed to the article and approved the submitted version.

## Funding

This work has been funded by Italian Ministry of Health, Ricerca Corrente program 2022−2024 to Gastroenterology Unit, Fondazione IRCCS “Casa Sollievo della Sofferenza” Hospital. Agorà Biomedical Sciences is a no-profit association committed to scientific research. Fundings for this manuscript came from the voluntary donations to the association.

## Conflict of interest

The authors declare that the research was conducted in the absence of any commercial or financial relationships that could be construed as a potential conflict of interest.

## Publisher’s note

All claims expressed in this article are solely those of the authors and do not necessarily represent those of their affiliated organizations, or those of the publisher, the editors and the reviewers. Any product that may be evaluated in this article, or claim that may be made by its manufacturer, is not guaranteed or endorsed by the publisher.

## Footnotes

^1^ http://www.ncbi.nlm.nih.gov/nuccore/AY234778.1

^2^ http://www.ncbi.nlm.nih.gov/nuccore/AY682469.2

^3^ https://blast.ncbi.nlm.nih.gov/

^4^ https://primer3.ut.ee/
